# DEC1 regulates breast cancer cell proliferation by stabilizing cyclin E protein and delays the progression of cell cycle S phase

**DOI:** 10.1038/cddis.2015.247

**Published:** 2015-09-24

**Authors:** H Bi, S Li, X Qu, M Wang, X Bai, Z Xu, X Ao, Z Jia, X Jiang, Y Yang, H Wu

**Affiliations:** 1 School of Life Science and Biotechnology, Dalian University of Technology, Dalian 116024, China; 2 School of Life Science and Medicine, Dalian University of Technology, Panjin 124221, China

## Abstract

Breast cancer that is accompanied by a high level of cyclin E expression usually exhibits poor prognosis and clinical outcome. Several factors are known to regulate the level of cyclin E during the cell cycle progression. The transcription factor DEC1 (also known as STRA13 and SHARP2) plays an important role in cell proliferation and apoptosis. Nevertheless, the mechanism of its role in cell proliferation is poorly understood. In this study, using the breast cancer cell lines MCF-7 and T47D, we showed that DEC1 could inhibit the cell cycle progression of breast cancer cells independently of its transcriptional activity. The cell cycle-dependent timing of DEC1 overexpression could affect the progression of the cell cycle through regulating the level of cyclin E protein. DEC1 stabilized cyclin E at the protein level by interacting with cyclin E. Overexpression of DEC1 repressed the interaction between cyclin E and its E3 ligase Fbw7*α*, consequently reducing the level of polyunbiquitinated cyclin E and increased the accumulation of non-ubiquitinated cyclin E. Furthermore, DEC1 also promoted the nuclear accumulation of Cdk2 and the formation of cyclin E/Cdk2 complex, as well as upregulating the activity of the cyclin E/Cdk2 complex, which inhibited the subsequent association of cyclin A with Cdk2. This had the effect of prolonging the S phase and suppressing the growth of breast cancers in a mouse xenograft model. These events probably constitute the essential steps in DEC1-regulated cell proliferation, thus opening up the possibility of a protein-based molecular strategy for eliminating cancer cells that manifest a high-level expression of cyclin E.

DEC1 belongs to a subfamily of bHLH transcription factors that are involved in a number of cell processes, including proliferation, apoptosis and circadian rhythms.^1, 2, 3, 4^ Several studies, which used protein overexpression and knockdown strategies have revealed the roles of DEC1 in cell cycle arrest, cell senescence and cell survival.^5^ Previous studies have shown that DEC1 can repress transcription in a HDAC-dependent manner, causing cell growth arrest, as well as demonstrating that DEC1 is a target of the p53 family and mediates cell cycle arrest and DNA damage-induced premature senescence.^6, 7, 8^ DEC1 also plays a role in cell survival. It mediates TGF-*β*-induced cell survival in breast cancer cells,^9^ and DEC1-overexpressing cells can resist oxidative stress-mediated cell death.^10^ Furthermore, DEC1 also regulates p53-dependent cell survival *versus* cell death through MIC-1 in response to DNA damage stress.^11^ Thus, DEC1 has multifaceted roles in cancer progression. However, whether it also affects cancer progression through regulating the cell cycle factors has not yet been clearly established.

Cyclin E, a member of the cyclin family, binds to and activates the Cdk2.^12^ The level of cyclin E protein oscillates throughout the cell cycle and peaks at around the beginning of the S phase, but subsequent degradation of the cyclin E protein is needed for the orderly cell progression to occur, which is regulated by E2Fs-dependent cyclin E transcription and ubiquitin-mediated cyclin E proteolysis.^13^ Two types of ubiquitin ligases are known to trigger the ubiquitin-mediated degradation of cyclin E, and these are the Cul1-(SCF) or Cul3-(BCR) dependent ubiquitin ligases.^14, 15, 16, 17^ Cyclin E that is bound to Cdk2 is targeted for ubiquitination by Cul1-dependent ubiquitin ligase, and this ubiquitination requires the phosphorylation of cyclin E at specific residues (Thr62, Ser372, Thr380 and Ser384).^12, 17, 18^ During the G_1_→S phase transition of the cell cycle progression, the formation of cyclin E/Cdk2 complex occurs in the nuclei and it needs to reach certain threshold in order to trigger the initiation of DNA replication.^7, 19^ However, abnormal stabilization of cyclin E inhibits transcription by increasing the initiation of replication and subsequently induces delay in the S phase.^20, 21^ Dysregulated activity of cyclin E is known to cause cell lineage-specific abnormalities such as impaired maturation as a result of increased genetic instability, cell proliferation and apoptosis or senescence via several different mechanisms.^16, 22^

In this study, we showed that DEC1 stabilized cyclin E without affecting its mRNA level. We also demonstrated that DEC1 stabilized cyclin E by blocking the proteasome pathway and hence, repressed the ubiquitination of cyclin E through reducing the interaction between cyclin E and Fbw7*α*. Furthermore, DEC1 promoted the activity and the formation of cyclin E/Cdk2 complex as well as the localization of cyclin E and Cdk2 in the nucleus, and repressed the subsequent formation of the cyclin A/Cdk2 complex, which led to the cells stalling at the S phase. These findings therefore provided new insight into the mechanism associated with DEC1-regulated cell cycle and proliferation of breast cancer cells.

## Results

### DEC1 expression is reduced in breast cancer cells and inhibits the proliferation

**Figure 1:**
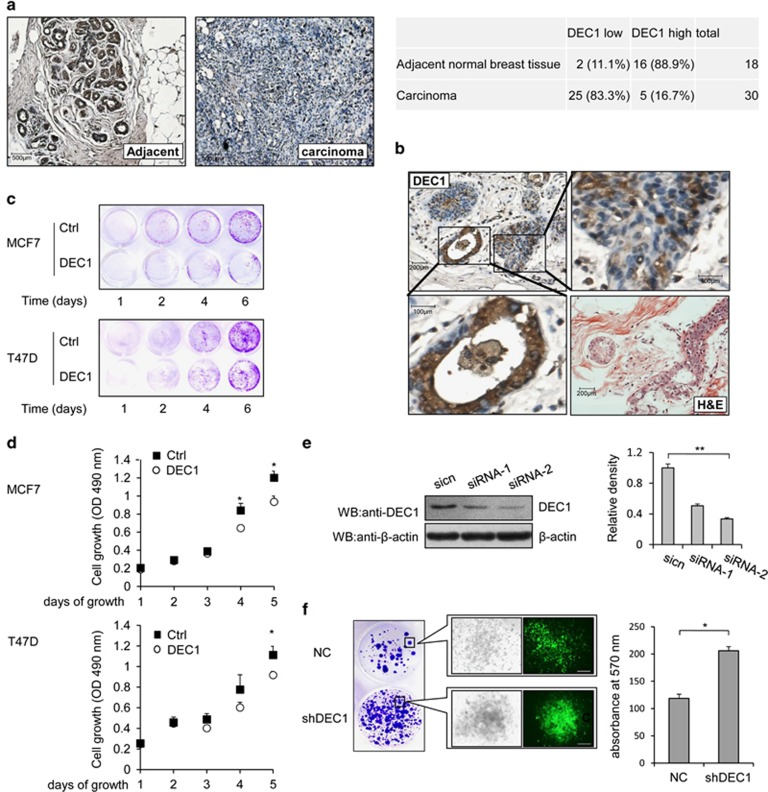
The expression of DEC1 in human breast tumor tissue samples and the effect of DEC1 on the cell proliferation. (**a** and **b**) Immunohistochemistry (IHC) analysis of DEC1 expression in adjacent normal breast tissues (*n*=18) and breast carcinoma (*n*=30). Each sample was incubated with antibody against DEC1. Positive staining and negative staining are indicated by brown and blue staining, respectively. (**c** and **d**) MCF-7 cells and T47D cells were transfected with Flag-DEC1 or control vectors (Ctrl). Cells were then cultured in selective medium (500 *μ*g/ml G418) and subjected to colony formation and MTT assays (days, time after transfection). Each bar represents the mean±S.D. from five independent experiments. *P<0.05. (**e**) Western blot analysis of endogenous DEC1 expression. MCF-7 cells were transfected with DEC1 siRNAs (siRNA-1 and siRNA-2) for 24 h and probed with anti-DEC1 and anti-*β*-actin. Each bar represents the mean±S.D. from three independent experiments. (**P<0.01). (**f**) Colony formation assay, MCF-7 cells were transfected with the shDEC1 vector, followed by 2-week selection with hygromycin B. Hygromycin-resistant clones are shown in the left panel. The right panel shows the corresponding quantitative analyses. Only representative data from three independent experiments are shown. Scale bar, 500 *μ*m

In order to study the function of DEC1 in breast cancer cells, we examined the expression and subcellular location of DEC1 in breast carcinoma and adjacent normal breast tissue using immunohistochemistry analysis. Overall, 25/30 (83.3%) of the breast carcinoma were negative for DEC1 and 16/18 (88.9%) of the adjacent normal breast tissue were positive for DEC1 (Figure 1a). DEC1 was highly expressed in adjacent noncancerous breast tissue, but only lowly expressed in breast carcinoma (Figures 1a and b; Supplementary Figure S1). The effect of DEC1 on the proliferation of breast cancer cells was investigated by overexpressing DEC1 in MCF-7 and T47D cells assessing their proliferation by MTT and colony formation assays. Overexpression of DEC1 inhibited the proliferation and colony formation of both MCF-7 and T47D cells (Figures 1c and d). Two DEC1 siRNAs (siRNA-1 and siRNA-2) were synthesized and used to knockdown the endogenous DEC1. siRNA-2 appeared to be more effective than siRNA-1 (Figure 1e). Therefore, the DEC1-specific target sequence within siRNA-2 was used to construct the shRNA (shDEC1) for the knockdown of DEC1 expression. The results showed that knockdown of DEC1 in MCF-7 cells increased the number and size of the colonies compared to the control cells (Figure 1f). These data suggested that DEC1 may play a regulatory role in the proliferation of MCF-7 and T47D breast cancer cells.

### DEC1 regulates cyclin E at late G1 phase and S phase

**Figure 2:**
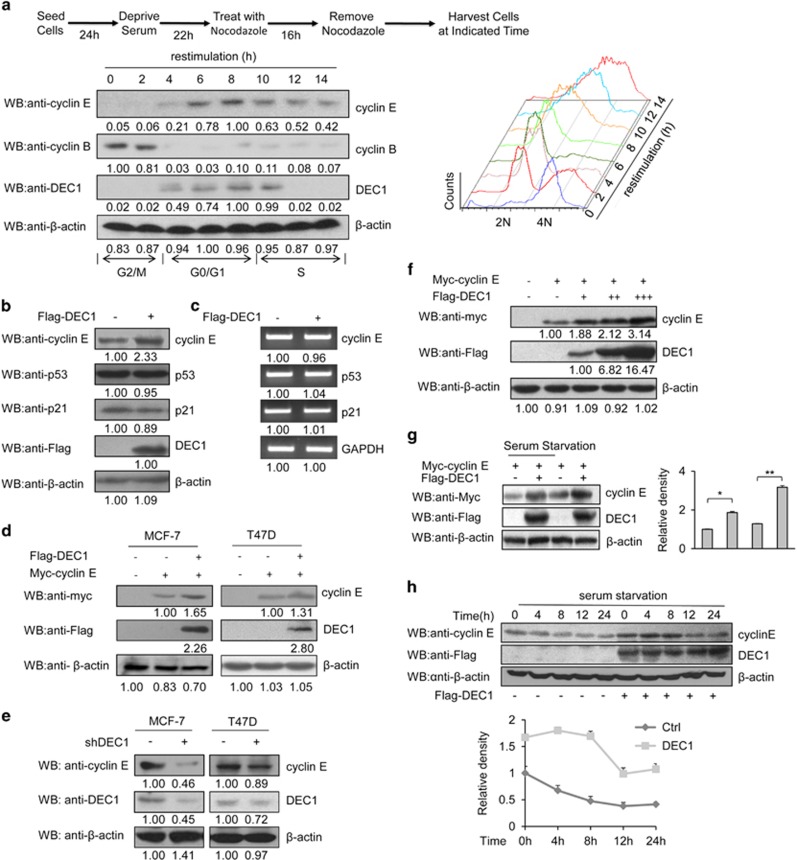
The expression dynamics of DEC1 in cell cycle and the effects of DEC1 expression on cyclin E stability. (**a**) Endogenous DEC1 levels during the cell cycle. MCF-7 cells were synchronized at G2/M by treating with 50 ng/ml nocodazole for 16 h. The cells were collected at the indicated time points following the removal of nocodazole. The cells were analyzed by FACS, and DEC1 level in the total cell lysate was determined by western blotting using anti-DEC1 antibody. (**b** and **c**) DEC1 affects the level of cyclin E protein but not in its transcription. The transcript and protein levels of cyclin E, p53 and p21 were measured in MCF-7 cells that overexpressed cyclin E only or cyclin E plus DEC1 by using reverse transcription PCR or by western blotting using specific antibodies against cyclin E, p53 and p21. (**d**) Western blot of cyclin E in DEC1-overexpressing T47D and MCF-7 cells. (**e**) T47D and MCF-7 cells transfected with DEC1 shRNA and probed with anti-DEC1 and anti-cyclin E antibodies. (**f**) DEC1 regulates cyclin E in a dose-dependent way. MCF-7 cells were transfected with Myc-tagged cyclin E and either empty vector or 0.5, 1 and 4 *μ*g Flag-DEC1. Lysates were analyzed by western blot with anti-myc, anti-Flag or anti-*β*-actin. (**g**) DEC1 regulates the level of cyclin E protein in both the presence and absence of serum. MCF-7 cells were transfected with the indicated plasmids for 12 h and were then starved for 24 h in serum-free medium. The histogram in the right plot shows the quantitative analysis of the bands. (**h**) Western blot analysis of the effect of DEC1 on the stability of cycin E under serum starvation condition. MCF-7 cells were transfected with or without DEC1 to detect the change in cyclin E protein level. The graph shows the relative intensity of the cycin E bands at different time points. In all experiments (**a**–**h**), *β*-actin expression or GAPDH mRNA level was used as a reference

To explore whether the levels of DEC1 protein at the various cell cycle stages are differently regulated, we measured the levels of DEC1 protein in MCF-7 cell extracts at every 2 h after 16 h of synchronization by nocodazole (which caused cell arrest at the G_2_/M stage; Figure 2a, upper panel). The phase of the cell cycle at each time point was determined by the expression profiles of cyclin B, cyclin E and FACS analysis (Figure 1a, bottom). The expression of endogenous DEC1 was increased during the late G_1_ phase, and peaked at the G_1_/S boundary before decreasing during the following S phase, which almost precisely overlapped with the expression dynamics of cyclin E (Figure 1a). These data suggested that DEC1 may play an important role in regulating the progression of cell cycle at the G_1_/S checkpoint.

We next investigated whether the involvement of DEC1 in the cell cycle progression would involve the regulation of these cell cycle factors (such as p53, p21 and cyclin E^23^) by DEC1. Surprisingly, overexpression of DEC1 led to an obvious increase in the level of endogenous cyclin E, but not of endogenous p53 protein, and a slight decrease in the level of endogenous p21 protein (Figure 2b) without affecting their mRNA levels (Figure 2c). Increased expression of cyclin E was induced by the overexpression of DEC1 and in a dose-dependent manner (Figures 2d and f). In contrast, knockdown of DEC1 decreased the level of endogenous cyclin E, especially in the case of MCF-7 cells (Figure 2e). Subsequent experiments that focused on the mechanism by which DEC1 could stabilize cyclin E were carried out in MCF-7 cells because the effect was more obvious in MCF-7 cells than in T47D cells. These data led us to hypothesize that DEC1 may regulate the expression of cyclin E independently of its transcriptional activity. To detect whether DEC1 could regulate the stability of cyclin E independently of its transcriptional activity, we constructed three truncated versions of DEC1 and tested if they could regulate the stability of cyclin E. Flag-DEC1 (302–412) contained neither the bHLH domain nor the three *α*-helices, and it could upregulate the stability of cyclin E (Supplementary Figures S2A and B). However, Flag-DEC1 (1–129) and Flag-DEC1 (129–301), which contained only the bHLH domain and the three *α*-helices, respectively, exhibited little or no effect on the protein level of cyclin E. This suggested that DEC1 may upregulate the stability of cyclin E independently of its transcriptional activity.

Since serum stress condition could block cell proliferation at the G_0_/G_1_ stage and decrease cyclin E expression,^24, 25^ we examined the role of DEC1 on the expression of cyclin E in MCF-7 cells that had been subjected to serum starvation, a condition that would effectively reduce the expression of cyclin E. DEC1 still upregulated the expression of cyclin E regardless of serum starvation (Figure 2g). Furthermore, the level of endogenous cyclin E was more stable in cells that overexpressed DEC1, but decreased sharply in the control cells, especially during the first 8 h of serum starvation (Figure 2h). These findings suggested that DEC1 was necessary for stabilizing cyclin E regardless of whether or not the cells were under serum starvation.

### DEC1 inhibits Fbw7-mediated cyclin E ubiquitin-proteasome pathway

**Figure 3:**
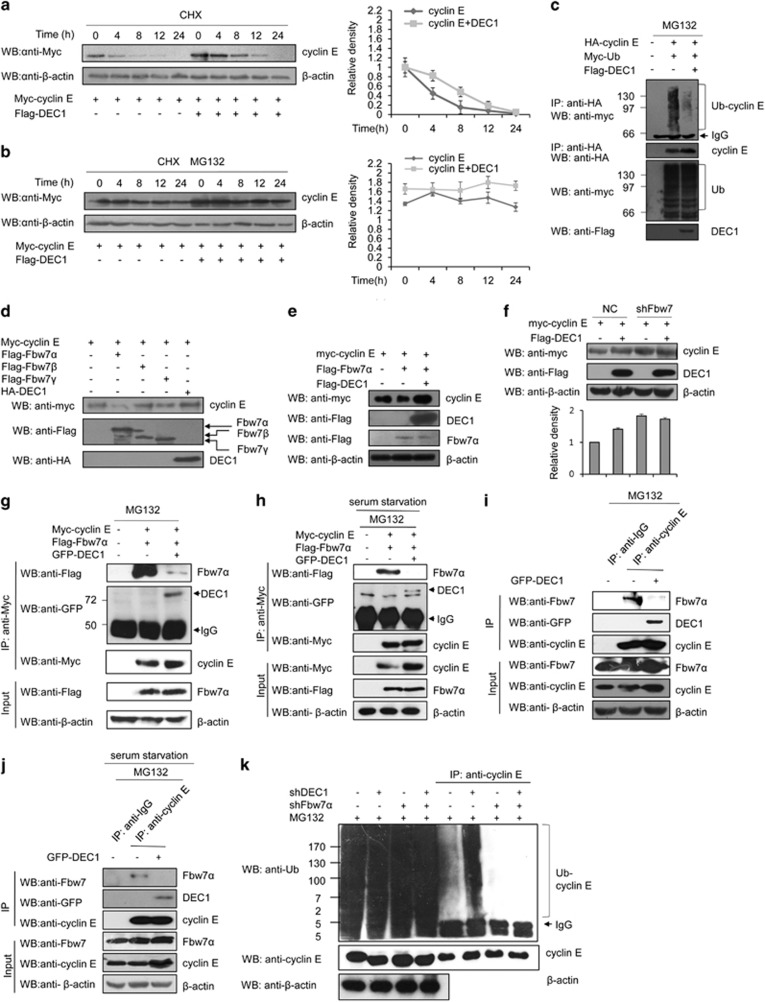
DEC1 regulates the stability of cyclin E in MCF-7 via Fbw7-mediated cyclin E ubiquitin-proteasome pathway. (**a** and **b**) Western blot analysis of the effect of DEC1 on the half-life of cyclin E. MCF-7 cells were transfected with Myc-cyclin E and Flag-DEC1 or empty vector. The cells were then treated with CHX only (**a**) or both CHX and MG132 (**b**), and harvested at the indicated time periods. The cell extract was subjected to western blot with anti-Myc, anti-Flag and anti-*β*-actin antibody. The histogram in the right panel (**a** and **b**) shows the quantitative analysis of cyclin E bands. (**c**) MCF-7 cells were transfected with HA-tagged cyclin E and Myc-Ub or empty vector, followed by treatment with MG132 for 8 h before harvest. The cell extract was immunoprecipitated with anti-HA antibody and then probed with anti-Myc antibody. (**d**) MCF-7 cells were transfected with plasmids expressing the indicated Flag-tagged Fbw7 isoforms together with Myc-tagged cyclin E or empty vector or DEC1, followed by western blot analysis with anti-Myc antibody. (**e**) MCF-7 cells were transfected with Myc-tagged cyclin E, Flag-Fbw7*α* and Flag-DEC1 or empty vector, followed by treatment with MG132. Clear cell extracts were probed with anti-Myc and anti-Flag antibodies. (**f**) MCF-7 cells were transfected with Myc-cyclin E only or together with Flag-DEC1 in the presence of sh-Fbw7 or sh-c. The histogram shows the quantitative analysis of cyclin E protein levels after normalization to *β*-actin (bottom). Data are the means±S.D. (**g**) MCF-7 cells were transfected with Myc-tagged cyclin E, Flag-Fbw7*α* and GFP-DEC1 or empty vector, treated with MG132 for 8 h before harvest. The clear cell extracts were immunoprecipitated with anti-Myc antibody, and then probed with anti-Flag, anti-GFP and anti-Myc antibody as indicated. *β*-Actin was used as a negative control. (**h**) Effect of DEC1 on the interaction between Fbw7*α* and cyclin E under serum starvation stress. MCF-7 cells were transfected as in (**g**), and cultured in serum-free medium, and then treated with 10 *μ*M MG132 for 8 h before harvest. The cells were collected and subjected to immunoprecipitation assay as in (**g**). (**i** and **j**) Effect of DEC1 on the interaction between endogenous Fbw7*α* and cyclin E. MCF-7 cells were transfected with GFP-DEC1 or empty vector, and cultured with or without serum, and then treated with MG132 for 8 h before harvest. The clear cell extracts were immunoprecipitated with anti-IgG or anti-cyclin E antibody, probed with anti-Fbw7, anti-GFP and anti-cyclin E antibody as indicated. *β*-Actin was used as a negative control. (**k**) Ubiquitination status of endogenous cyclin E in DEC1- and Fbw7-silenced cells. MCF-7 cells were transfected with either shDEC1 or shFbw7*α*, or both. Cell extract was immunoprecipitated with anti-cyclin E antibodies, followed by western blotting using anti-Ub or anti-cyclin E antibody. Total cell lysate was also analyzed by western blotting using anti-*β*-actin antibody. To test the expression and immunoprecipitation of cyclin E, mouse monoclonal (SB62a) secondary antibody against rabbit IgG light chain (HRP, ab99697) was used at a 1 : 5000 dilution in (**g**–**k**)

The positive effect that DEC1 exerted on the stability of cyclin E suggested that it might increase the half-life of cyclin E. Indeed, DEC1 markedly extended the half-life of cyclin E from 4 to 8 h in MCF-7 (Figure 3a) and T47D (Supplementary Figure S3B) cells. Increase in the half-life of a protein usually involves a reduction of its degradation via the proteasome pathway.^26^ When the cells were treated with CHX plus the proteasome inhibitor MG132, overexpression of DEC1 did not enhance the half-life of cyclin E (Figure 3b) suggesting that DEC1 may regulate the stability of cyclin E protein via the ubiquitin-proteasome pathway. Subsequent ubiquitination assay showed that in the absence of DEC1 overexpression, cyclin E was heavily ubiquitinated (Figure 3c, lane 2), whereas in the presence of DEC1 overexpression, cyclin E ubiquitination was markedly decreased (Figure 3c, lane 3). Since Fbw7s is the ubiquitination E3 ligase of cyclin E,^17^ the effects of all three isoforms of Fbw7s on the stability of cyclin E were investigated. Cyclin E level was most significantly affected by Fbw7*α*, which caused the highest reduction compared to the other two isoforms (Figure 3d). However, this effect of Fbw7*α* on cyclin E was compromised by DEC1, since cells that overexpressed Fbw7*α*, cyclin E and DEC1 yielded the same level of cyclin E as cells that overexpressed cyclin E only (Figure 3e). When Fbw7s in the cells was knocked down by shFbw7, overexpression of DEC1 exhibited no increase in the level of cyclin E compared to cells in which Fbw7s was also knocked down, but without overexpression of DEC1 (Figure 3f). Since the effect of DEC1 on the stability of cyclin E depended on Fbw7*α*, we sought to determine whether DEC1 could affect the interaction between cyclin E and Fbw7*α*. Co-immunoprecipitation experiment showed that overexpression of DEC1 markedly reduced the interaction between Fbw7*α* and cyclin E whether or not the cells were under serum starvation (Figures 3g–j). In addition, we also investigated the ubiquitination of endogenous cyclin E in the cells in which either DEC1 or Fbw7 had been knocked down as well as in the cells in which both DEC1 and Fbw7 had been knocked down (Figure 3k). This demonstrated that the inhibition of cyclin E ubiquitination by DEC1 was dependent on the presence of Fbw7. Taken together these results indicated that DEC1 stabilized cyclin E protein through blocking the ubiquitin-mediated proteasomal degradation of cyclin E, which probably occurred through a reduction of interaction between cyclin E and Fbw7*α*.

### DEC1 is a cyclin E-interacting protein

**Figure 4:**
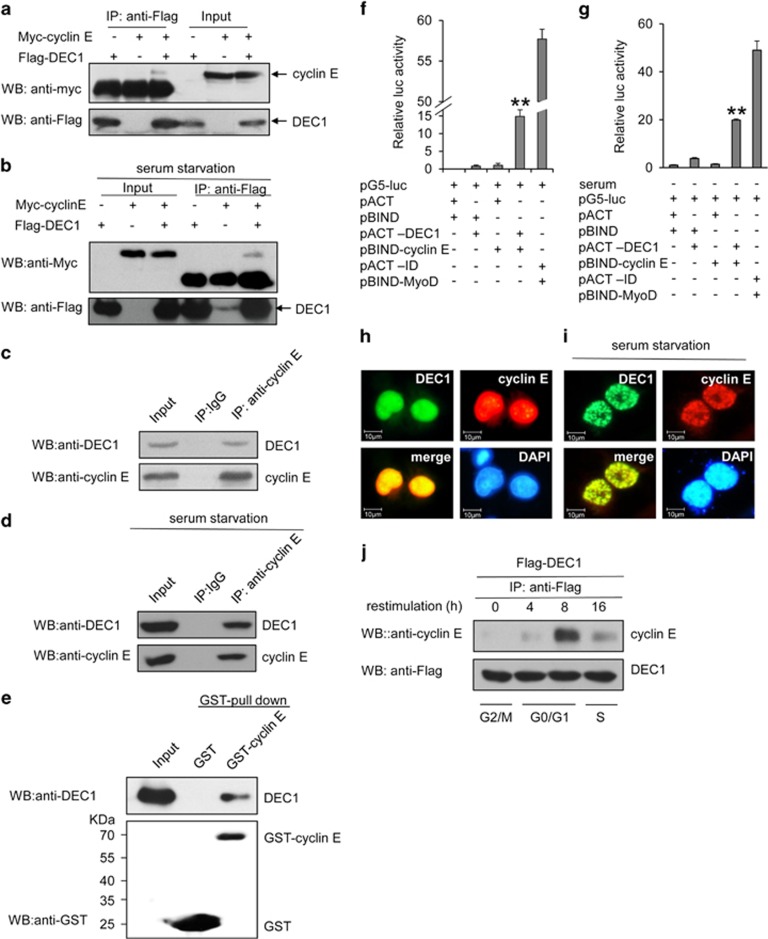
Interaction between DEC1 and cyclin E. (**a**) MCF-7 cells were transfected with Flag-tagged DEC1 and Myc-tagged cyclin E. After 24 h of transfection, the cell extract was subjected to immunoprecipitation with anti-Flag antibody, followed by western blot analysis with anti-Myc antibody. (**b**) MCF-7 cells transfected as in (**a**) were subjected to serum starvation for 24 h. The cell extract was subjected to immunoprecipitation with anti-Flag antibody followed by western blot analysis with anti-Myc antibody. (**c** and **d**) CoIP experiment showing the interaction between endogenous DEC1 and cyclin E in serum-plus and serum-free conditions. MCF-7 cells were subjected to immunoprecipitation with anti-cyclin E antibody followed by western blot analysis with anti-DEC1 antibody. Immunoprecipitation carried out with anti-IgG antibody was used as control. Mouse monoclonal (SB62a) secondary antibody against rabbit IgG light chain (HRP) was used at a 1 : 5000 dilution. (**e**) Western blot analysis of GST pull-down assay showing the interaction between cyclin E and DEC1. The cell extract was incubated with glutathione-agarose beads coated with purified GST or GST-tagged cyclin E as indicated at the top. After extensive washing, bound proteins were eluted, resolved by SDS-PAGE, and probed with anti-DEC1 and anti-GST antibodies. (**f** and **g**) Interaction between DEC1 and cyclin E as detected by a mammalian two-hybrid system for cells without and with serum starvation. Cyclin E and DEC1 were expressed from pBIND-cyclin E and pACT-DEC1, respectively. The MCF-7 cells were transfected with pG5-luc and the empty vectors (pACT and pBIND) as indicated. Positive control cells were transfected with pBIND-ID and pACT-MyoD. Each bar represents the mean±S.D. from three independent experiments. **P<0.01 compared with cells transfected with pACT and pBIND. (**h** and **i**) Co-localization of DEC1 and cyclin E in MCF-7 cell nuclei. MCF-7 cells were transfected with Myc-cyclin E and Flag-DEC1. Flag-antibody complex and Myc-antibody complex were visualized with FITC and TRITC, respectively. Nuclei were stained with 4,6-diamidino-2-phenylindole (DAPI). (**j**) CoIP experiments showing the dynamics of the interaction between DEC1 and cyclin E. MCF-7 cells were transfected with Flag-DEC1, and then subjected to immunoprecipitation with anti-Flag antibody followed by western blot analysis with anti-cyclin E antibody. HRP (ab99697) was used as secondary antibody

The positive regulation of cyclin E by DEC1 at the protein level suggested that these two proteins might interact with each other. Co-immunoprecipitation experiment showed that DEC1 specifically interacted with cyclin E in MCF-7 cells regardless of whether or not the cells were under serum starvation (Figures 4a and b). The interaction between endogenous DEC1 and cyclin E was also verified in MCF-7 cells, both in the absence and presence of serum (Figures 4c and d). GST pull-down assay and the CheckMate mammalian two-hybrid system (Promega, Madison, WI, USA) also detected the association of cyclin E with DEC1 (Figures 4e–g). Furthermore, DEC1 and cyclin E exhibited a pronounced nuclear co-localization (Figures 4h and i). This indicated the presence of a substantial interaction between DEC1 and cyclin E.

We then explored the dynamics of the interaction between DEC1 and cyclin E at different stages of the cell cycle. The result showed that the interaction of these two protein displayed cell cycle-dependent dynamics, detected mainly at the G_1_/S phase 8 h after release from nocodazole treatment (Figure 4j). These data also suggested that DEC1 may play an important role in the regulation of the cell cycle progression by interacting and regulating the activity of cyclin E at the G_1_ and S phases.

### DEC1 promotes the cyclin E/Cdk2 complex formation

**Figure 5:**
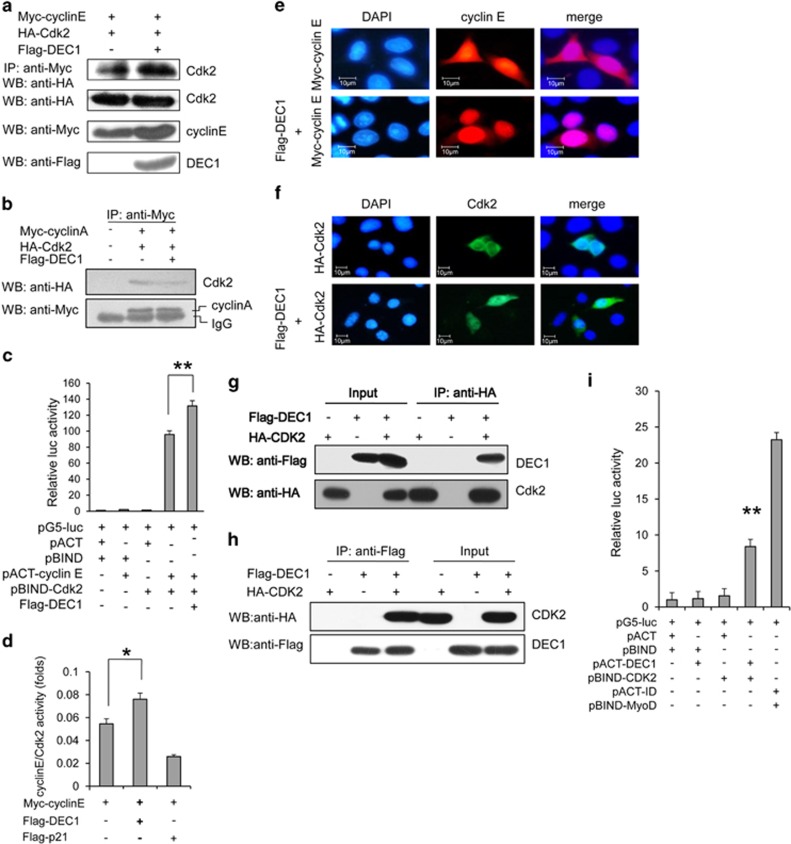
DEC1 promotes the complex formation and activity of cyclin E/Cdk2 and inhibits cell cycle progression through the S phase. (**a**) Effect of DEC1 on the formation of cyclin E/Cdk2 complex. MCF-7 cells were transfected with HA-Cdk2 and Myc-cyclin E only or HA-Cdk2, Myc-cyclin E and Flag-DEC1, and the clear cell extract was immunoprecipitated with anti-Myc antibodies and then probed with anti-HA antibody. (**b**) Effect of DEC1 on the sequent formation of the cyclin A/Cdk2 complex as detected by CoIP. MCF-7 cells were transfected with empty vector only, HA-Cdk2 and Myc-cyclin A only or HA-Cdk2, Myc-cyclin A and Flag-DEC1, and the clear cell extract was immunoprecipitated with anti-Myc antibody and then probed with anti-HA antibody. (**c**) Mammalian two-hybrid assay analysis of the effect of DEC1 on the interaction between cyclin E and Cdk2. Cyclin E, Cdk2 and DEC1 were expressed from pBIND-cyclin E, pACT-Cdk2 and Flag-DEC1, respectively. Each bar represents the mean±S.D. from three independent experiments. **P<0.01 compared with cells transfected with pACT and pBIND. (**d**) Effect of DEC1 on the activity of Cdk2/cyclin E kinase. Columns, mean (*n*=3)±S.D.; *P<0.05 compared with controls. (**e**) Immunofluorescence assay showing the effect of DEC1 on the sublocation of cyclin E. MCF-7 cells were transfected with Myc-cyclin E only or Myc-cyclin E and Flag-DEC1, and were fixed and stained with rabbit anti-Myc antibody (red) and then counterstained with DAPI (blue) for nucleus detection. (**f**) Effect of DEC1 on the sublocation of Cdk2. MCF-7 cells were transfected with HA-Cdk2 only or HA-Cdk2 and Flag-DEC1 and stained with mouse anti-HA antibody (green), and then counterstained with DAPI (blue) for nucleus detection. (**g** and **h**) CoIP assay showing the interaction between DEC1 and Cdk2. Control cells or cells overexpressing HA-Cdk2 only, Flag-DEC1 only or Flag-DEC1 and HA-Cdk2 were subjected to immunoprecipitation with anti-HA antibody (**g**) and with anti-Flag antibody (**h**). (**i**) Mammalian two-hybrid assay analysis of the interaction between DEC1 and Cdk2. DEC1 and Cdk2 were expressed from pACT-DEC1 and pBIND-Cdk2, respectively. Columns, mean (*n*=3)±S.D.; **P<0.01 compared with controls

Like all cyclin family members, cyclin E forms a complex with cyclin-dependent kinase (Cdk2) at the G_1_/S phase checkpoint, which needs to be degraded, and this will in turn promote the formation of cyclin A/Cdk2 complex and activity during the transition from S to G_2_ phase.^27, 28, 29^ Immunoprecipitation assay showed that DEC1 increased the amount of cyclin E-associated Cdk2 and decreased the interaction between cyclin A and Cdk2 by ~50% (Figures 5a and b). This effect of DEC1 was also confirmed by the CheckMate mammalian two-hybrid system (Promega), which showed that cells that overexpressed DEC1 displayed a significant increase in reporter activity over cells that overexpressed cyclin E and Cdk2 (Figure 5c). Furthermore, the level of cyclin E/Cdk2 kinase activity of cells that overexpressed cyclin E and DEC1 was significantly higher than the level of those that either overexpressed cyclin E or p21 (the Cdk2 inhibitor used as a negative control) alone (Figure 5d). Since, cyclin E functions as a nuclear protein, and is associated with Cdk2 during the G1/S phase transition,^30^ we tested whether DEC1 would affect the sublocalization of cyclin E and Cdk2. As shown in Figures 5e and f, cyclin E and Cdk2 were localized in both the cytoplasm and the nucleus, but in the presence of DEC1, they were predominantly localized in the nucleus. These data suggested that overexpression of DEC1 may affect the progression of cell cycle S phase.

By using co-immunoprecipitation assay and mammalian two-hybrid assay, we showed that DEC1 could bind to cdk2, but without affecting the level of Cdk2 (Figures 5g–i and Supplementary Figure S4E). These data suggested that a high level of cyclin E (induced by DEC1) may stabilize the interaction between cyclin E and Cdk2, consequently interfering with the association between Cdk2 and cyclin A, an event that could lead to the cells stalling at the S phase.

### Overexpression of DEC1 causes defect in cyclin E degradation, results in cell cycle S-phase delay

**Figure 6:**
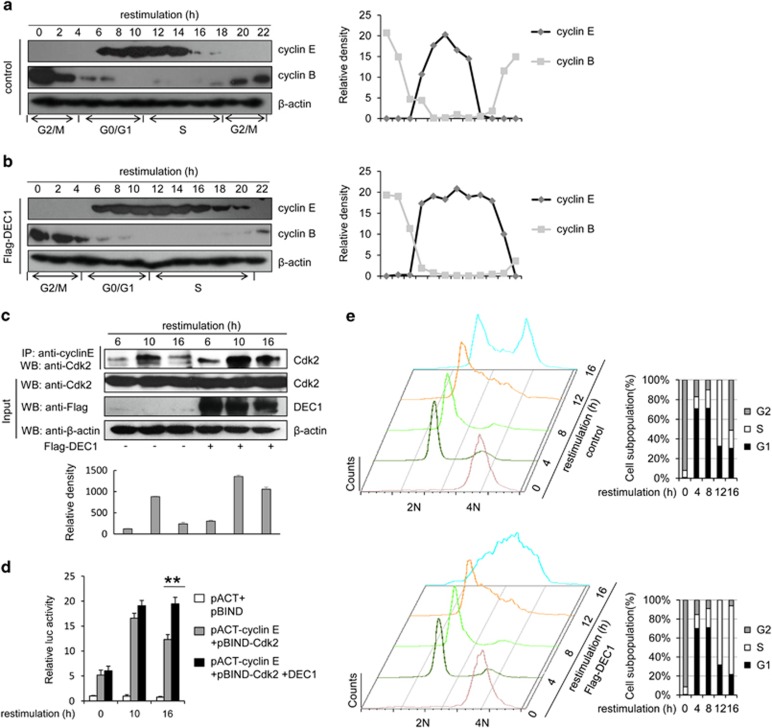
DEC1 inhibits the progression of S phase in the cell cycle. (**a** and **b**) Western blot of cyclin E and cyclin B in control and DEC1-overexpressing cells. The plots show the relative intensities of the bands in the blots. MCF-7 cells were transfected with empty vector or Flag-DEC1, and then synchronized at G2/M stage by treating with 50 ng/ml nocodazole for 20 h. The cells were analyzed at different time points after release from nocodazole treatment. The clear cell extract was subjected to western blot analysis with anti-cyclin E, anti-cyclin B or anti-*β*-actin antibody. (**c**) MCF-7 cells were treated as in (**a** and **b**) and then subjected to immunoprecipitation using anti-cyclin E antibody followed by western blot with anti-Cdk2, anti-DEC1 and anti-*β*-actin antibody. (**d**) Effect of DEC1 on the dynamics of cyclin E and Cdk2 as demonstrated by mammalian two-hybrid assay. Cyclin E, Cdk2 and DEC1 were expressed from pACT-cyclin E, pBIND-Cdk2 and Flag-DEC1, respectively. MCF-7 cells were transfected with the indicated plasmids, synchronized and harvested at the indicated time points. Each bar represents the mean±S.D. from three independent experiments. **P<0.01 compared with cells transfected with pACT and pBIND. (**e**) Effect of DEC1 on the cell cycle progression (on the left panel) as demonstrated by FACS assay. Quantified analysis is shown by histogram in the right panel. MCF-7 cells were transfected with DEC1 or control vector, synchronized and harvested at the indicated time points

We next synchronized MCF-7 to specifically study the effect of DEC1 on cell cycle from early G_2_/M phase to late S phase. We noticed that in the cells that expressed DEC1, the level of cyclin E persisted for four more hours while the level of cyclin B was delayed for 4 h compared to the levels of the control cells. This suggested that overexpression of DEC1 could extend the S phase of the cells, raising the possibility that DEC1-induced increased stability of cyclin E could inhibit the progression of the cells through the S phase (Figures 6a and b). To further confirm this possibility, we performed co-immunoprecipitation (CoIP) experiment and found that the interaction between cyclin E and Cdk2 was clearly increased at the G_1_/S transition checkpoint (from 6 to 10 h after release) and decreased at the late S phase (from 16 h after release; Figure 6c and Supplementary Figure S4A). This observation was further verified by the mammalian two-hybrid system (Figure 6d). However, in DEC1-overexpressing cells, the stabilized cyclin E remained associated with Cdk2 and inhibited the subsequent formation of cyclin A/Cdk2 complex 16 h after release from nocodazole treatment (Figure 6c and Supplementary Figure S4B).

Flow cytometric analysis of the synchronized cells indicated that DEC1-overexpressing cells had an extended S phase compared to the control (Figure 6e and Supplementary Figure S4C). Delay in cell cycle may have a substantial negative effect on the genome stability and replication initiation.^21^ We found that cells overexpressing DEC1 had higher level of *γ*H2AX than control cells, and in a cyclin E-dependent manner (Supplementary Figure S4D). Taken together, these results demonstrated that DEC1 delayed cell cycle progression through the S phase and induced genome instability, eventually resulting in repression of cell proliferation.

### DEC1 inhibits the proliferation of cells overexpressing cyclin E and inhibits tumor xenograft growth

**Figure 7:**
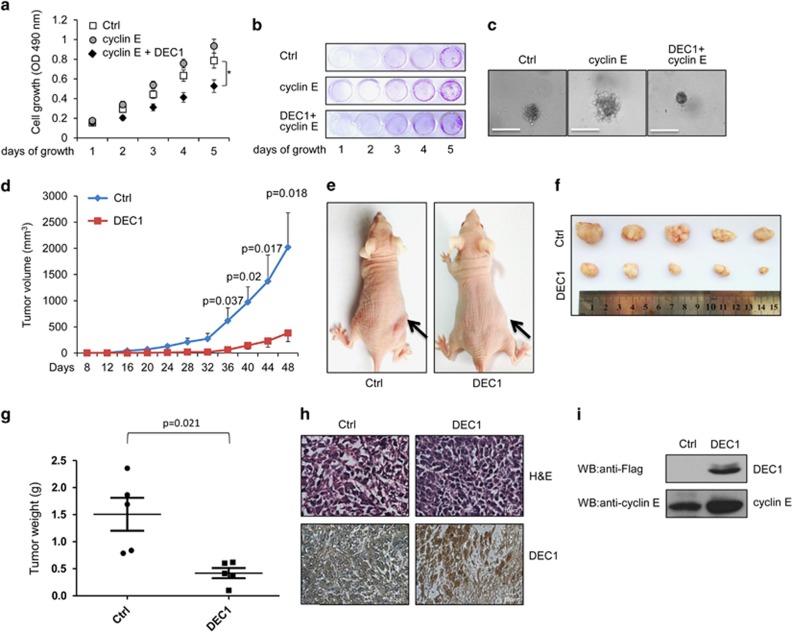
DEC1 inhibits the proliferation of cells overexpressing cyclin E and inhibits tumor xenograft growth. (**a** and **b**) MCF-7 cells were transfected with Myc-cyclin E only, Myc-cyclin E and Flag-DEC1 or control vector. The cells were then cultured in selective medium (500 *μ*g/ml G418) and subjected to colony formation and MTT assays. Each bar represents the mean±S.D. from five independent experiments. *P-value was determined by ANOVA with Bonferroni test (*P<0.05). (**c**) Representative colonies of each experimental group are shown. MCF-7 cells transfected with control vector, Myc-cyclin E or Myc-cyclin E and Flag-DEC1 were selected with 800 *μ*g/ml G418 for 15 days. The cells were then collected and suspended in a soft agar. Photographs of the colonies were taken 30 days after seeding. Scale bar, 100 *μ*m. All experiments were repeated at least three times. (**d**) Tumor growth by subcutaneously implanted MCF-7 cells (6 × 10^5^ cells) transfected with pCMV10-3 × Flag or pCMV10-3 × Flag-DEC1 and screened with G418. *P*-value was determined by unpaired *t*-test. (**e**) Tumor formation 36 days after the mice were injected with the tumor cells. Left: mice injected with control cells. Right: mice injected with DEC1 overexpressed cells. (**f** and **g**) Tumor images (**f**) and tumor weight (**g**) 48 days after mice were injected subcutaneously with MCF-7 cells overexpressed Flag-DEC1 or empty vector. *n*=5 mice per group in (**d**, **f** and **g**). (**h**) Representative immunohistochemical data for H&E and DEC1 on paraffin-embedded section of subcutaneous tumors in (**f**) and (**g**), generated by MCF-7 cells overexpressed DEC1 or empty vector. (**i**) Western blot analysis of the expression of cyclin E and DEC1 in control (Ctrl) and Flag-DEC1-overexpressing tumors

The effect exerted by DEC1 on the growth of cyclin E-overexpressing MCF-7 cells was examined. As expected, cells that overexpressed cyclin E had a high growth rate than cells that did not overexpress DEC1 (transfected with empty vector), but the growth rate of MCF-7 cells that overexpressed both DEC1 and cyclin E became partly inhibited over a 5- day period (Figure 7a). Moreover, colony formation assay and soft agar assay showed that cells that overexpressed both DEC1 and cyclin E produced significantly lower number of colonies as well as smaller colonies compared to cells that only overexpressed cyclin E (Figures 7b and c). These results indicated that DEC1 may be used as an inhibitor or suppressor for tumor growth.

To further investigate the effect of DEC1 on tumorigenicity *in vivo*, we subcutaneously implanted MCF-7 cells that stably overexpressed DEC1 or those that harbored the empty vector into nude mice and monitored the size of the tumor developed from these cells. Mice that were implanted with DEC1-overexpressing MCF-7 cells showed a much smaller tumor throughout the experimental period than mice implanted with MCF-7 cells that harbored the empty vector (Figures 7d and f). Forty-eight days after tumor cell implantation, a 2.6-fold decrease in the weight of the tumors was achieved for MCF-7 cells that overexpressed DEC1 (Figure 7g). Examination of the expression level of DEC1 in the tumor by IHC and WB showed that DEC1 was successfully expressed in tumors with small sizes and the level of cyclin E in the tumor was also upregulated (Figures 7h and i; Supplementary Figure S6). Overall, these *in vitro* and *in vivo* experiments indicated that DEC1 functioned as a tumor suppressor and inhibited cell growth.

## Discussion

As an important transcription factor, DEC1 plays an important role in cell differentiation, proliferation and apoptosis.^31, 32, 33, 34, 35^ In this study, we found in this study that DEC1 could affect the level of cyclin E in a cell, not through its transcriptional activity (Figures 2b and c; Supplementary Figures S2A and D), but through protein-protein interaction, and this effectively allowed DEC1 to regulate the cell cycle progression, consequently resulting in the regulation of cell proliferation. As shown in Figures 2 and 3, DEC1 upregulated the level of cyclin E protein in a dose-dependent pathway and prolonged the half-life of cyclin E, and the underlying mechanism by which it achieved this was through interfering with the interaction between cyclin E and Fbw7, thereby reducing the Fbw7-mediated ubiquitination of cyclin E (Figures 3g–j). In addition to increasing the protein level of cyclin E through inhibiting the Fbw7-mediated ubiquitination and degradation pathway, DEC1 also decreased the level of p21 (Supplementary Figure S5) and enhanced the binding of cyclin E to Cdk2 as well as the kinase activity of the cyclin E/Cdk2 complex (Figures 5a and d), suggesting that multiple mechanisms could be at work.^36^

More and more evidences have shown that cyclin E may function as a ‘switch' or a double-edged sword: however, high expression of cyclin E promotes a faster transition from G_1_ to S phase,^37, 38^ which is why cyclin E is always expressed at a high level in various types of cancers and its expression correlates with tumorigenesis;^39, 40^ however, excessive cyclin E would interfere with the assembly of the pre-replication complex and lead to replication stress, DNA damage and genomic instability, which will block the S-phase progression and cause cell cycle arrest.^21, 41, 42^ In this study, we have verified the following: (i) DEC1 could promote the nuclear accumulation of cyclin E/Cdk2 and inhibit the formation of cyclin A/Cdk2, which would cause the cells to stay in the S phase resulting in cell cycle arrest (Figures 5 and 6). (ii) DEC1 could interact with Cdk2 directly without influencing the level of Cdk2, which may work as a platform to enhance the formation of cyclin E and Cdk2. (iii) Overexpression of DEC1 would increase the level of *γ*H2AX in cells subjected to serum-deprived stress (S4D), and this may cause genetic instability. However, we could not yet determine whether the high level of *γ*H2AX in the cells that overexpressed DEC1 reflected increased DNA damage, impaired DNA repair, or both. (iv) A high level of DEC1 expression in adjacent normal breast tissue compared to the carcinoma (Figure 1a). We speculated that DEC1 may not only regulate cell proliferation but may also be involved in metastasis, which is also a subject for further study.

In addition, although DEC1 could interact with cyclin E both in the presence and absence of serum, noticeable difference in the localization of cyclin E-DEC1 between the two conditions was detected (Figures 4h and i). The co-localization of DEC1 and cyclin E in the nucleus was more obvious under serum starvation condition than under normal culturing conditions. Thus, DEC1 could potentially be involved in the binding of MCM proteins to the replication origin by interacting with cyclin E under serum starvation condition.

In summary, our data demonstrated that DEC1 promoted the formation of cyclin E/Cdk2 complex and inhibited the subsequent formation of cyclin A/Cdk2 complex leading to the stalling of S phase of the cell cycle and inhibition of cell proliferation. This valuable insight would help us to conceive a way to combat cancers; especially those mediated by cyclin E dysregulation, and may even provide a unique therapeutic strategy to control the growth of these cancer cells.

## Materials and Methods

### Cell culture and transfection

MCF-7 and T47D cells have been used in our previous study and were maintained in our laboratory as standard human breast cancer cell lines.^43, 44^ The cells were cultured in DMEM (Invitrogen, Carlsbad, CA, USA) supplemented with 10% FBS (Hyclone, Beijing, China), 100 mg/ml penicillin and 100 mg/ml streptomycin at 37 °C in the presence of 5% CO_2_. For synchronization, the cells were cultured in DMEM without FBS for 22 h. After that FBS was added to the culture to a final concentration of 10%, and the culture was incubated for 2 h before the addition of nocodazole (Sigma, St. Louis, MO, USA) to a final concentration of 50 ng/ml. After 16 h of incubation, the cells were washed twice with complete DMEM medium.^45^ For ubiquitination assay, the cells were treated with 10 *μ*M MG132 (Sigma) for 8 h before harvest. For protein half-life assay, the cells were treated with 50 *μ*M CHX alone or together with MG132 after transfection. Transfection of the cells was carried out using Lipofectamine 2000 (Invitrogen) according to the manufacturer's instruction.^46^

### Plasmids, siRNA and antibodies

pTB701-HA/cyclin E was provided by Mikiko Takahashi (Biosignal Research Center, Kobe University, Kobe, Japan). pCS^2+^-Myc/cyclin E harboring wild-type and mutant forms (T380A and T380S mutants) of cyclin E were acquired from Clurman Lab (Fred Hutchinson Cancer Research Center, Seattle, WA, USA). p3xFlag-CMV7.1/Fbw7*α* was provided by Dr. Deanna M Koepp (University of Minnesota-Twin Cities, Minneapolis and St. Paul, MN, USA). HA-Cdk2 was kindly given by Dr. Greg H. Enders (Fox Chase Cancer Center, Philadelphia, PA, USA). pCMV-Myc/cyclin A was provided by Dr. Liang Zhu (Department of Developmental and Molecular Biology, Jack and Pearl Resnick Campus, Bronx, NY, USA). The plasmids p3xFlag-CMV-10/DEC1, p3xFlag-CMV-10, pcDNA3.1-Myc/Ub, pcDNA3.1(−) and pcDNA3.1-HA/DEC1 were acquired as previously described.^47, 48^ PCR primers for constructing siRNAs, pRNAT-U6.1/shDEC1, pACT/DEC1, pACT/cyclin E, pBIND/cyclin E and pBIND/Cdk2 are presented in Supplementary Table S1.

Antibodies used in this study included anti-Myc (9E10), -Flag (M2; Sigma); anti-DEC1 (A300-649A; Bethyl Laboratories, Montgomery, TX, USA); anti-Ub (10201-2-AP; Proteintech, Wuhan, China); anti-Fbw7 (A301-720A-T; Bethyl Laboratories); anti-Cdk2 (ab6538; Abcam, Cambridge, UK); anti-cyclin B1 (AB60234a; Sango, Shanghai, China); anti-HA (sc-7392), -cyclin E (sc-198), -cyclin A (sc-751), -mouse and -rabbit secondary antibodies (Santa Cruz Biotechnology, Dallas, TX, USA); mouse monoclonal (SB62a) antibody against rabbit IgG light chain (HRP, ab99697; Abcam).

### Immunoblotting and protein-protein interaction

Cells were lysed in 200 *μ*l lysis buffer (50 mM Tris-HCl pH 8.0, 150 mM NaCl, 0.1% SDS, 1% NP-40 and 0.5% sodium deoxycholate), and centrifuged at 12 000 × *g*/4 °C for 10 min to obtain the cell extract.^49, 50^ The protein concentration of the cell extract was determined before subjecting to western blot or immunoprecipitation analysis. For immunoblotting, aliquot of this cell extract was first resolved in 10% SDS-PAGE gel, and the protein bands were then transferred to Millipore (Billerica, MA, USA) PVDF membranes.

For CoIP assay, the cell extract was incubated with the appropriate primary antibody and protein A-Sepharose (Amersham Biosciences, Piscataway, NJ, USA) at 4 °C for 24 h, followed by centrifugation at 5000 × *g*/4 °C for 10 min. The pellet was washed twice with wash buffer I (50 mM Tris-HCl pH 7.5, 500 mM sodium chloride, 0.1% NP-40 and 0.05% sodium deoxycholate) and once with wash buffer II (50 mM Tris-HCl pH 7.5, 0.1% NP-40 and 0.05% sodium deoxycholate), and then subjected to SDS-PAGE using 10% gel followed by western blot.^51^ Immunoblot data were quantified by scanning the appropriate bands of interest and plotted as relative density of gray scale. The CheckMate mammalian two-hybrid system (Promega) was used according to manufacturer's protocol. The primers used are shown in Supplementary Table S1.

### GST pull-down assay

GST and GST-tagged cyclin E-fusion proteins were expressed in *Escherichia coli* BL21(DE3) and purified with the Pierce GST spin purification kit (Thermo scientific, Waltham, MA, USA) according to the manufacturer's instructions. The identity of the purified protein was confirmed by western blot with an anti-GST antibody. The purified GST-tagged fusion protein (BAIT) was immobilized on the Pierce spin column. MCF-7 cells were lysed in pull-down lysis buffer containing DNase (Takara, Dalian, China) and centrifuged at 12 000 × *g*/4 °C for 15 min to obtain the cell extract. The supernatant was loaded onto the Pierce spin column, and then incubated at 4 °C for at least 6 h with gentle agitation. After that the column was centrifuged at 700 × *g* for 1 min and the flow through was discarded. The column was then washed five times with wash solution and centrifuged as before with each wash. After the last wash, elution buffer was added to the column and the column was incubated for 5 min with gentle agitation, before it was centrifuged at 700 × *g* for 1 min. The resulting eluent was subjected to western blot assay.

### Cell cycle analysis

MCF-7 were trypsinized, fixed in 70% ethanol at 4 °C, and washed according to previously described protocol.^52^ The washed cells were incubated in PBS containing propidium iodide and RNase A at 37 °C for 30 min and then subjected to cell cycle analysis performed with a Becton Dickinson FACScan run by the FlowJo 7.6 software (BD Biosciences, San Jose, CA, USA).^53^

### Cell proliferation assays

Cell proliferation was determined by MTT assay and colony formation assay. For MTT assay, cells (5 × 10^4^ per well) were seeded into 96-well plate and incubated with appropriate selective medium for several days. After that, MTT assay was performed according to the manufacturer's protocol (KeyGen, Nanjing, China).

For colony formation assays, the cells were transfected with the appropriate plasmids and then transferred to 35-mm plate containing the appropriate selective medium and incubated for different times. After that, the cells were fixed in cold 75% ethanol for 10 min and dyed with 30% crystal violet (Sigma) for 15 min at room temperature. The cells were then washed with water to remove the residual crystal violet, and the number of individual colonies was counted.

### Soft-agar colony culture

Anchorage-independent growth of MCF-7 cells was estimated using soft agar colony formation assay as described previously.^54^ The cells were transfected with the appropriate plasmids and the same number of cells from each transfection were then cultured in selective medium containing 1 × DMEM, 10% FBS and 800 *μ*g/ml G418 (Sigma). After 15 days of culturing, the cell were collected and 6 × 10^4^ cells were suspended in 1 ml of 0.35% soft agar medium (top agar pre-warmed to 37 °C), which was then added to the top of a 0.6% agar base layer (prepared in 1 ml medium) in a 35-mm-diameter dish. Fresh medium (200 *μ*l, containing 200 *μ*g/ml G418) was added to the plate 3 days later and on every successive 3 days over a 30-day period. The colonies that appeared in the plate were counted and photographed using a phase-contrast microscope, and the diameters of the colonies was measured the with the software Image-Pro Plus 6.0 (Media Cybernetics, Rockville, MD, USA).^55^

### Immunofluorescence staining

MCF-7 cells were grown on cover slips for overnight, and then transfected with the desired plasmids. Twenty-four hours after transfection the cells were washed three times with PBS, fixed in 4% paraformaldehyde for 15 min at room temperature, permeabilized with 0.1% Triton X-100 in PBS solution at −20 °C, and finally blocked with 0.8% BSA for 1 h at 4 °C. The cells were then incubated with the corresponding antibodies and examined according to the manufacturer's instructions.

### Kinase activity assay

The kinase activity of cyclin E/Cdk2 complex was measured with a kinase activity assay kit (Genmed Scientifics, Arlington, MA, USA), which is a complete assay system designed to measure the activity of cyclin E/Cdk2 by coupling the formation of ADP to the reaction catalyzed by PK and LDH in the presence of phosphoenolpyruvate, which would result in the oxidation of NADH. The disappearance of NADH was detected by measuring the decrease in absorbance at 340 nm at every 1-min interval over a 5-min period. The assay was repeated three times for each sample.

### Human breast cancer xenograft model

Five- to six-week-old athymic BALB/c nude male mice were purchased from the Animal Experiment Center of Dalian Medical University and maintained under specific pathogen-free (SPF) conditions (NO.SCXK2013-0003). All experiments were carried out according to the regulation set by the Ethics Committee for Biology and Medical Science of Dalian University of Technology. About 6 × 10^5^ MCF-7 cells stably transfected with Flag-DEC1 or empty plasmid (as selected by G418) were resuspended in a final volume of 100 *μ*l containing 50% Matrigel (BD Matrigel, BD Biosciences) and then injected into the right flanks of the animals (*n*=5 mice per group). Tumor growth rates were analyzed by caliper measurements every 8 days post injection using the formula: tumor volume (*V*)=length (*L*) × width (*W*)^2^ × 0.5. After 48 days, the animals were killed in a humane manner and the tumors were weighed, photographed and subjected to further analysis (paraffin embedding and digestion for subsequent extraction of protein) as previously described.^56^

### Luciferase reporter assays

Cells were seeded in 24-well plates at a density of 2 × 10^5^ per well and cultured for 24 h before they were transfected with the appropriate plasmids. Twenty-four hours after transfection, the cells were harvested and Luc reporter assay was performed according to the manufacturer's instructions (Promega).

### Statistical analysis

All statistical analyses of data were performed with ANOVA with LSD method. Data were expressed as means±S.D., and significance was considered at either *P*-value <0.05 or 0.01 level.^54^

## Abbreviations

DEC1: differentiated embryo-chondrocyte expressed gene 1
bHLH: basic helix-loop-helix
HDAC: histone deacetylase
MIC-1: macrophage inhibitory cytokine 1
TGF: transforming growth factor
E2F: adenovirus E2 factor
Cdk: cyclin-dependent kinase
Cul: cullin
SCF: Skp1-Cul1-F-box protein complex
BCR: BTB-Cul3-Rbx1 complex
FACS: fluorescence-activated cell sorting
miRNA: microRNA
shRNA: short hairpin RNA
MCM: minichromsome maintenance
DMEM: Dulbecco's modified Eagle's medium
FBS: fetal bovine serum
MTT: 3-(4,5-dimethyl-2-thiazolyl)-2,5-diphenyl-2-H-tetrazolium bromide
CHX: cycloheximide
