# Supplementary material for: DEC1 regulates breast cancer cell proliferation by stabilizing cyclin E protein and delays the progression of cell cycle S phase
Source: Cell Death Dis. 2015 Sep 24;6(9):e1891–. doi: 10.1038/cddis.2015.247 (PMC4650443; doi:10.1038/cddis.2015.247)
Supplement: Supplementary Information [file cddis2015247x1.doc]

**Table S1 Primers for RT-PCR and DEC1 shRNA construction and Mammalian Two-Hybrid System (MTH)**

| **Primer name** | **Sequence** |
| --- | --- |
| DEC1 siRNA-1-sense | 5’-r (CCAAAGUGAUGGACUUCAA)d (TT)-3' |
| DEC1 siRNA-1-antisense | 5’-r (UUGAAGUCCAUCACUUUGG) d(GA)-3' |
| DEC1 siRNA-2-sense | 5’-r (GCACUAACAAACCUAAUUG) d (TT)-3’ |
| DEC1 siRNA-2-antisense | 5’-r (CAAUUAGGUUUGUUAGUGC) d(GA)-3’ |
| shDEC1-a | 5’-GATCCC**GCACTAACAAACCTAATTG**TTCAAGAGA **CAATTAGGTTTGTTAGTGC**TTTTTTCCAAA-3’ |
| shDEC1-b | 5’-AGCTTTTGGAAAAAA**GCACTAACAAACCTAATTG** TCTCTTGAA**CAATTAGGTTTGTTAGTGC**GG-3’ |
| p53-RT-F | 5’-GCTGCTCAGATAGCGATGG-3’ (sense) |
| p53-RT-R(293 bp) | 5’-AGGACAGGCACAAACACG-3’ (antisense) |
| p21-RT-F | 5’-TTGATTAGCAGCGGAACA-3’ (sense) |
| p21-RT-R(270 bp) | 5’-TACAGTCTAGGTGGAGAAACG-3’ (antisense) |
| cyclin E-RT-F | 5’-GGATGTTGACTGCCTTGA-3’ (sense) |
| cyclin E-RT-R(354 bp) | 5’-CTGCTCTGCTTCTTACCG-3’ (antisense) |
| GAPDH-RT-F | 5’- TGAAGGTCGGAGTCAACGG -3’ (sense) |
| GAPDH-RT-R(225 bp) | 5’- CCTGGAAGATGGTGATGGG -3’ (antisense) |
| CDK2-MTHS-F | 5’- CGG ATC CGT ATG GAG AAC TTC CAA AAG G-3’ |
| CDK2-MTHS-R | 5’-GGATATCTCA GAG TCG AAG ATG G-3’ |
| DEC1-MTHS-F | 5’-CGACGCGTTA ATG GAG CGG ATC-3’ |
| DEC1-MTHS-R | 5’-GCTCTAGA TTA GTC TTT GGT TT-3’ |
| cyclin E-MTHS-F | 5’-CGACGCGTTA CCG AGG GAG C-3’ |
| cyclin E-MTHS-R | 5’-GCTCTAGATCA CGC CAT TTC CG-3’ |

DEC1 and CDK2 were inserted into pACT and pBIND at the *Mlu*Ⅰ-*Xba*Ⅰand *BamH*Ⅰ-*EcoR*Ⅴ sites, respectively. Cyclin E was inserted into pACT and pBIND at the *Mlu*Ⅰ-*Xba*Ⅰsite.

**Fig. S1. DEC1 is expressed at low level in breast carcinoma.**

Low magnification of Fig. 1B showing the immunohistochemistry (IHC) analysis of DEC1 expression in breast carcinoma. Scale bar, 1000 μm.

**Fig. S2 Effects of** **different truncations of DEC1 on the proliferation of cell cycle that depends on stabilizing cyclin E.**

(A) Schematic representation of the Flag-DEC1 different truncations. The various domains of DEC1 are shown. bHLH, basic helix-loop-helix; H1, helix 1; H2, helix 2; H3, helix 3.

(B) MCF-7 cells were transfected with Myc-cyclin E and either empty vector or Flag-DEC1 and subjected to western blot with anti-Myc, anti-Flag or anti-β-actin antibody.

(C) MCF-7 cells were transfected with Myc-cyclin E only, Myc-cyclin E and Flag-DEC1 truncations or control vector. The cells were then cultured in selective medium (500 μg/ml G418) and subjected to MTT assays. Each bar represents the mean ± S.D. from five independent experiments. * *P* value was determined by ANOVA with Bonferroni test (*, *P* < 0.05).

(D) Representative colonies from soft agar colony formation assay are shown for of each experimental group. MCF-7 cells as Fig. S2C and were selected with 800 μg/ml G418 for 15 days. The cells were then collected and suspended in a soft agar. Photographs of the colonies were taken 30 days after seeding. Scale bar, 100 μm.

**Fig. S3. DEC1 up-regulated the protein levels of cyclin E in cos-7 cells.**

(A) Western blot of cyclin E in DEC1-overexpressed cos-7 cells. Lysates were analyzed by western blot with anti-GFP, anti-Flag or anti-β-actin.

(B) Western blot analysis of the effect of DEC1 on the half-life of cyclin E. Cos-7 cells were transfected with GFP-cyclin E and Flag-DEC1 or empty vector, and then treated with CHX at the indicated time periods. Cell extracts were subjected to western blot analysis with anti-GFP or anti-β-actin antibody.

**Fig. S4. DEC1 delays cell cycle progression through S phase.**

(A-B) MCF-7 cells were transfected with empty vector or Flag-DEC1, synchronized at G2/M by treating with nocodazole then released and collected at the indicated time points. The clear cell extracts were subjected to immunoprecipitation using anti-cyclin A antibody (B), or anti-cyclin E antibody (A) followed by western blot with anti-CDK2 or anti-DEC1 antibody.

(C) FACS assay of the effect of DEC1 on the cell cycle progression. MCF-7 cells were transfected with DEC1 or control vector, synchronized by serum starvation and harvested at the indicated time points.

(D) MCF-7 cells transfected with Flag-DEC1 only, shcyclin E only or Flag-DEC1 and shcyclin E, and then serum deprived for 48 hours. The cells were fixed and immunostained for γH2AX (Red) and DAPI (blue). Quantitative analysis of γH2AX relative intensities is shown (Right panel). Data are the means ± SDs.

(E) Effect of DEC1 on the stability of CDK2. MCF-7 cells were transfected with HA-CDK2 and either empty vector or 0.5, 1, and 4 μg Flag-DEC1. Lysates were analyzed by western blot with anti-HA, anti-Flag or anti-β-actin.

**Fig. S5 DEC1 down-regulates the level of p21 protein.**

(A) Western blot analysis of the effect of DEC1 on the half-life of p21. MCF-7 cells were transfected with Flag-p21 and Flag-DEC1 or empty vector, and then treated with CHX at the indicated time periods. Cell extracts were subjected to western blot with anti-Flag or anti-β-actin antibody.

(B) DEC1 regulates p21 in dose-dependent way. MCF-7 cells were transfected with Flag-p21 and either empty vector or 0.5, 1, and 4 μg Flag-DEC1. Lysates were analyzed by western blot with anti-Flag or anti-β-actin.

**Fig. S6 DEC1 up-regulates the protein level of full length cyclin E but not low-molecular-weight (LMW) cyclin E in the tumor.**

Western blotting analysis of the whole unedited blot of Figure 7i, which shows the expression of the cyclin E and DEC1 in control (Ctrl) and Flag-DEC1-overexpressing tumors.
