# Supplementary figures and images for: DEC1 regulates breast cancer cell proliferation by stabilizing cyclin E protein and delays the progression of cell cycle S phase
Source: Cell Death Dis. 2015 Sep 24;6(9):e1891–. doi: 10.1038/cddis.2015.247 (PMC4650443; doi:10.1038/cddis.2015.247)

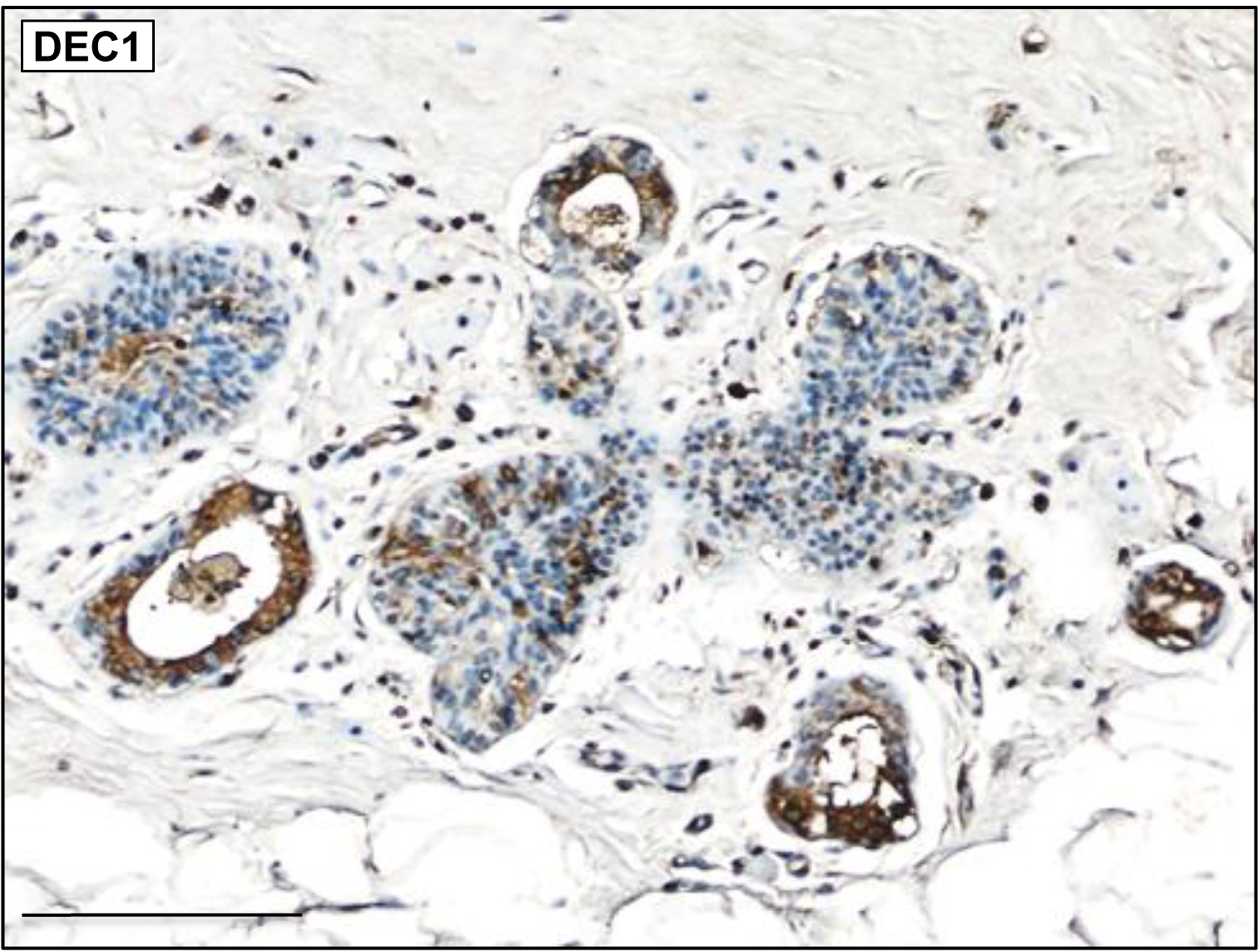

Supplement: Supplementary Figure 1 [file cddis2015247x2.tif]

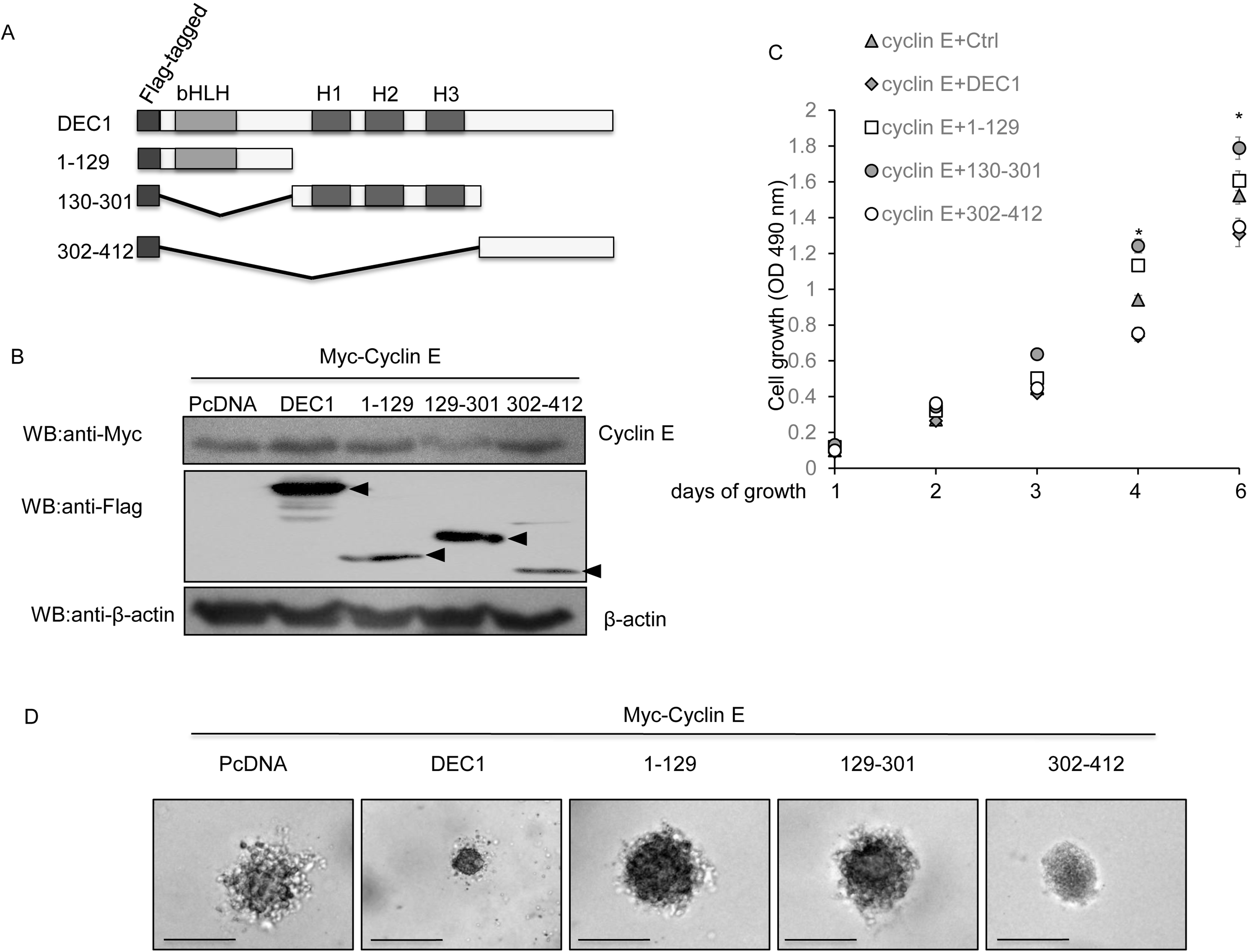

Supplement: Supplementary Figure 2 [file cddis2015247x3.tif]

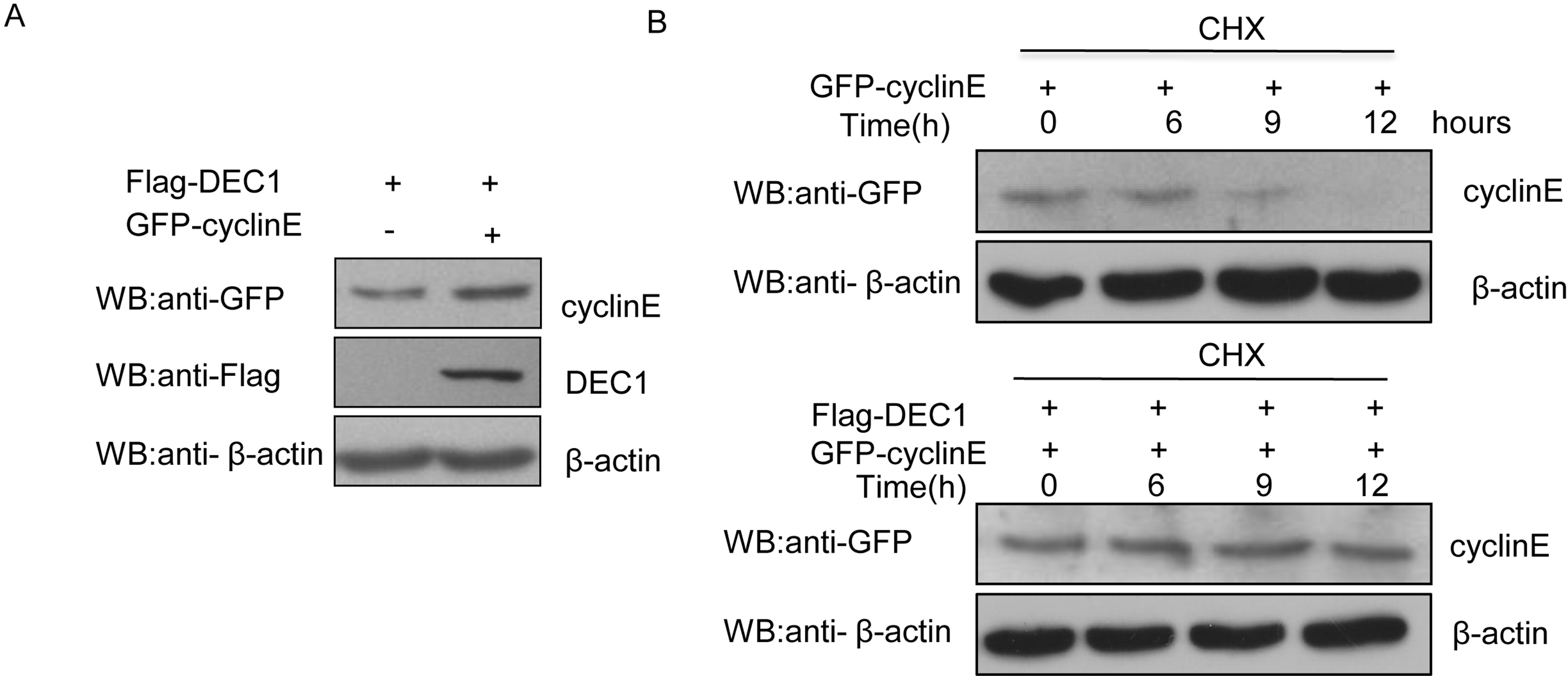

Supplement: Supplementary Figure 3 [file cddis2015247x4.tif]

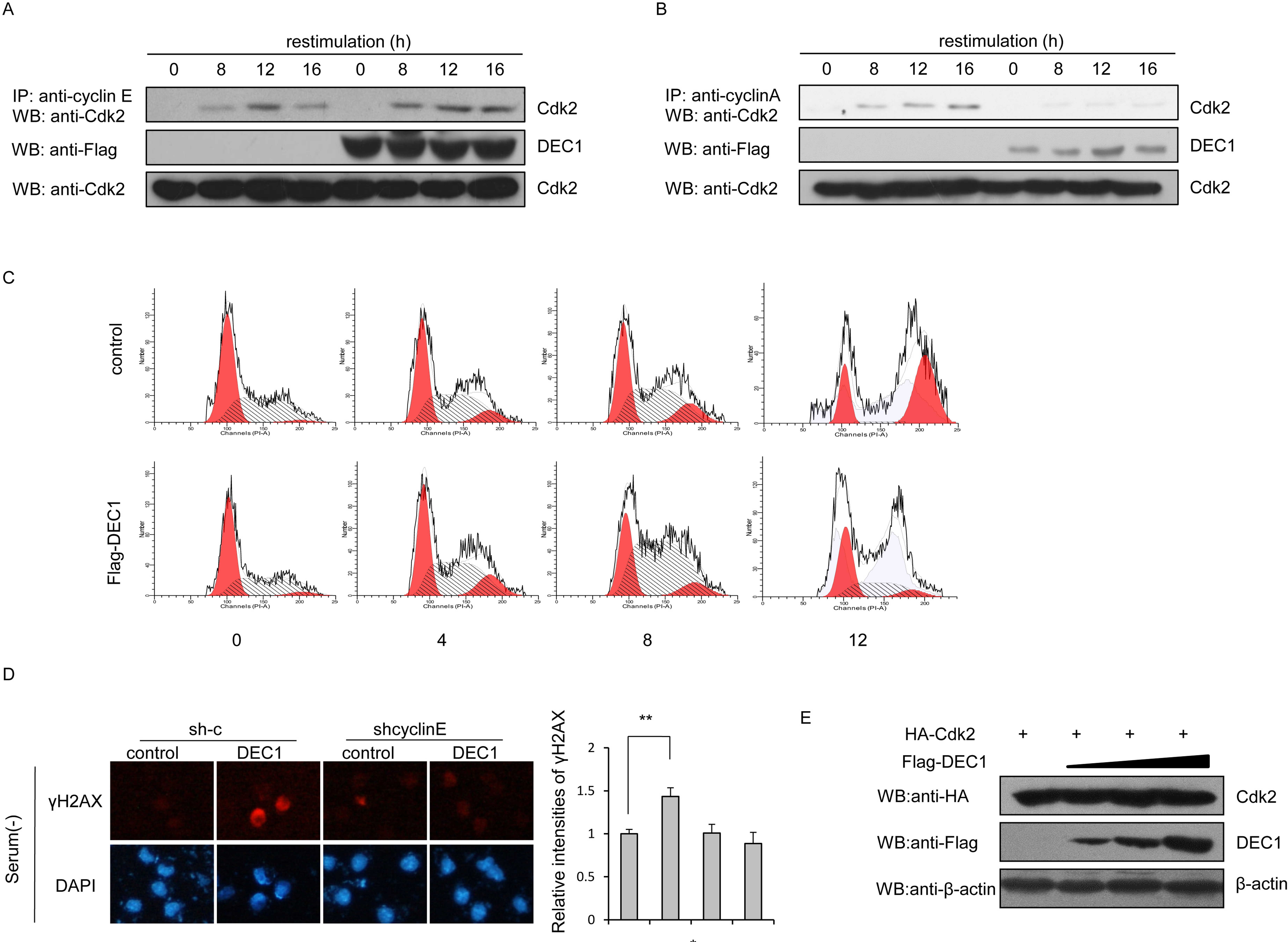

Supplement: Supplementary Figure 4 [file cddis2015247x5.tif]

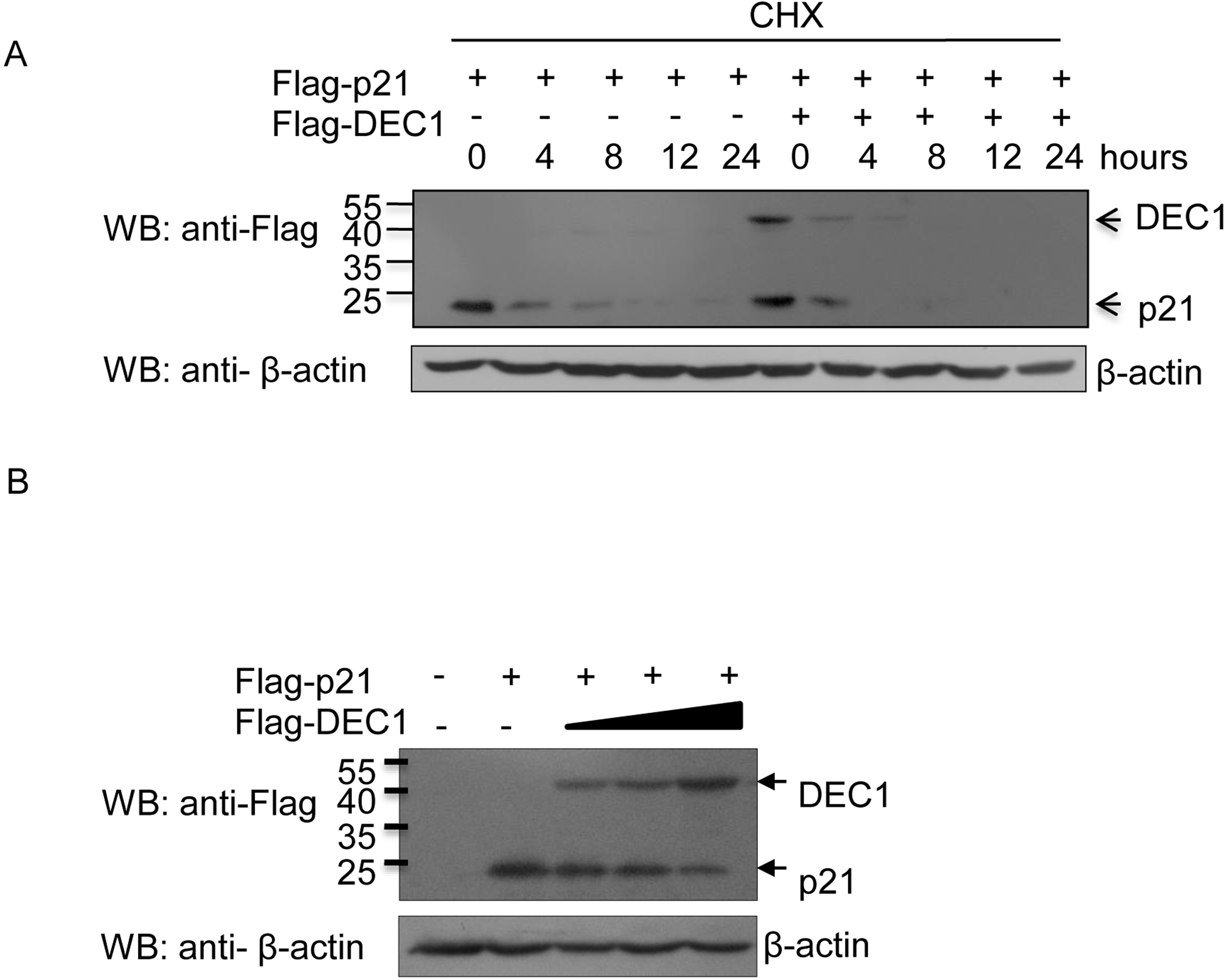

Supplement: Supplementary Figure 5 [file cddis2015247x6.tif]

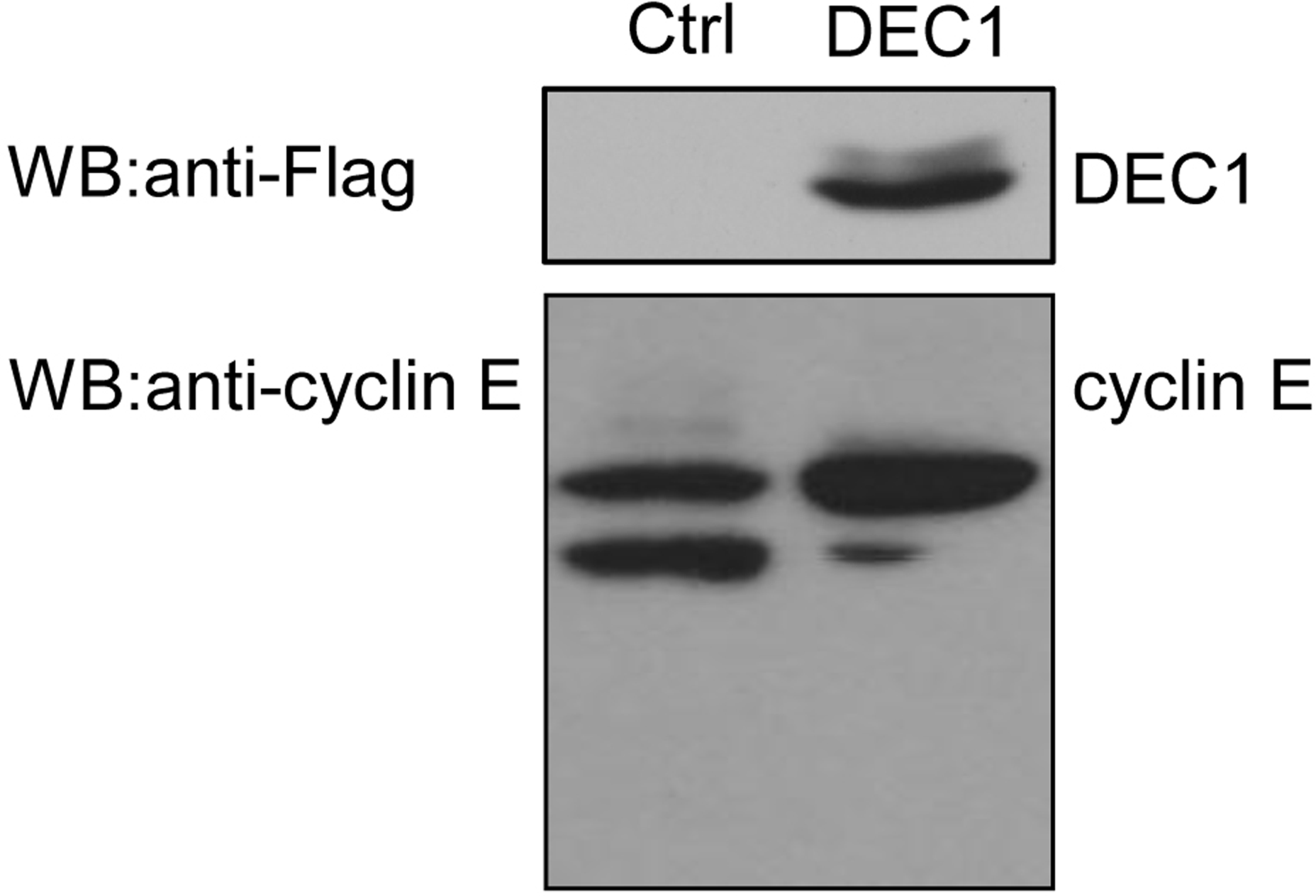

Supplement: Supplementary Figure 6 [file cddis2015247x7.tif]
